# Effects of a multispecies synbiotic on intestinal mucosa immune responses

**Published:** 2019-08

**Authors:** Alpha Fardah Athiyyah, Nur Aisiyah Widjaja, Pramira Fitri, Ariani Setiowati, Andy Darma, Reza Ranuh, Subijanto Marto Sudarmo

**Affiliations:** Department of Child Health, Dr. Soetomo General Hospital, School of Medicine, Universitas Airlangga, Surabaya, Indonesia

**Keywords:** Synbiotic, *Lactobacillus*, *Bifidobacterium*, *Streptococcus*, Immune response

## Abstract

**Background and Objectives:**

Probiotics and prebiotics are known to regulate immune responses. A synbiotic is a product that combines probiotics and prebiotics in a single dosage form. In this study, we attempt to present the effects of a multispecies synbiotic on intestinal mucosa immune responses after exposure to *Escherichia coli* O55:B5 lipopolysaccharide (LPS).

**Materials and Methods:**

Totally 21 male Balb/c mice were randomly classified into two groups. The K-I group received LPS and a synbiotic, and the K-II group received LPS alone. The synbiotic was administered for 21 consecutive days, whereas LPS was administered once on the 15^th^ day. Specifically, a synbiotic containing 1 × 10^9^ colony forming units (CFUs) of the probiotic combination of *Lactobacillus acidophilus* PXN 35, *L. casei* subsp. *casei* PXN 37, *L. rhamnosus* PXN 54, *L. bulgaricus* PXN 39, *Bifidobacterium breve* PXN 25, *B. infantis* PXN 27 and *Streptococcus thermophilus* PXN 66 and the prebiotic fructo-oligosaccharide was administered through an orogastric tube. Immunohistochemistry was performed to measure immunoglobulin A (IgA) levels for humoral immune responses and CD4+ and CD8+ levels for cellular immune responses.

**Results:**

An independent-samples t-test revealed significant increases of the numbers of IgA- (p = 0.027) and CD4-expressing cells (p = 0.009) but not the number of CD8-expressing cells in the K-I group compared with those in the K-II group.

**Conclusion:**

The multispecies synbiotic had immunoregulatory effects on IgA and CD4 expression in LPS-exposed mice.

## INTRODUCTION

The mucosal immune system is an important part of the immune system. About 90% of the infection process occurs in the mucosa, primarily in the gastrointestinal mucosa (1, 2). As a digestive organ, the intestine possesses the widest mucosal surface in the entire body; hence, it is more easily exposed to the outside environment, which is rich in antigens derived from commensal bacteria and pathogens, viruses and food antigens. To confer protection against this continuous exposure, the intestinal mucosa can produce local immune responses because it contains immunocompetent and immunosecretory cells (3–5).

Innate (natural) and adaptive (produced) immunity are two essential elements of the immune defence mechanism (6). Antigens, which are identified as pathogens, are captured through the tight junctions of enterocytes within the lumen by antigen-presenting cells (macrophages and dendritic cells) with the help of Toll-like receptor (TLR), which is a component of innate immunity. TLR functions as a pattern-recognition receptor in mammals that plays an important role in the introduction of non-self pathogen components (such as bacteria, viruses, fungi and parasites) and recognises endogenous ligands that appear during the acquisition of inflammatory responses (7, 8). This pathogen is presented by both B cells and immature T cells. Pathogens presented by B lymphocytes trigger the transformation of mature B cells into plasma cells, which produce immunoglobulins (humoral immunity). The introduction of pathogens to T lymphocyte receptors is performed by HLA class I, which generally presents endogenous antigens, and HLA class II, which primarily presents exogenous antigens. HLA class I triggers cytotoxic cellular responses, which are largely controlled by CD8+ (cytotoxic T) lymphocytes, whereas HLA class II triggers cellular responses, which are controlled by CD4+ T lymphocytes (T helper) (9, 10).

A synbiotic combines microorganisms demonstrated (or believed) to have beneficial effects when consumed (i.e. a probiotic) and a compound that specifically favours their growth (i.e. a prebiotic), with the combination having a synergistic effect. Many probiotic supplements are currently marketed as synbiotics (11). Synbiotics have multiple and different influences on the host. They have antimicrobial activity, which can reduce intestinal lumen pH, induce antimicrobial peptide secretion, inhibit bacterial invasion and bacterial adhesion towards epithelial cells, improve barrier function by increasing mucus production, increase barrier integrity and stimulate immunomodulation, in several cell types including epithelial cells, dendritic cells, monocytes/ macrophages and lymphocytes (B lymphocytes, NK cells, T cells) (12, 13). Research on mice and humans prove that probiotics can induce an immune response and accelerate the reversal of both acute and chronic gastrointestinal disorders (4, 14, 15). However, the effects of synbiotics on the production of immunoglobulin A (IgA), CD4 and CD8 in the intestinal mucosa of healthy and lipopolysaccharide (LPS)-exposed mice have not been studied. This study aimed to examine the effects of synbiotics on the regulation of humoral immune responses, represented by IgA-expressing cell counts, and adaptive immune responses, represented by CD4- and CD8-expressing cell counts, in the intestinal mucosal in mice.

## MATERIALS AND METHODS

### Animals.

Balb/c mice (n = 21), age, 10–12 weeks; weight, 30–40 g, (Veterinaria Farma Centre, Surabaya) were used. Ethics approval was obtained from the Ethics Committee (Animal Care and Use Committee) of Veterinary Medicine School. Animals were housed for 1 week and daily fed. Mice were randomised into synbiotic + LPS (K-I) and LPS (K-II) groups, 10 mice each. The K-I group mice received the synbiotic daily during the study (21 consecutive days) and LPS from *Escherichia coli* O55:B5 (L2880; Sigma-Aldrich, St. Louis, MO, USA) once (on day 15) via a gastric tube, whereas K-II group mice received LPS only (on day 15) in the same manner. The ileum of each animal was dissected for analysis at the end of experiment. Daily examination were conducted for the symptoms of illness, such as reduced activity, abnormal evacuation and decreased body weight.

### Synbiotics and LPS.

This study used synbiotics contained 1 × 10^9^ CFUs of a combination of probiotics, i.e. *L. bulgaricus* PXN 39, *L. casei* subsp. *casei* PXN 37, *Bifidobacterium breve* PXN 25, *L. rhamnosus* PXN 54, *B. infantis* PXN 27 *Lactobacillus acidophilus* PXN 35, *Streptococcus thermophilus* PXN 66, and the prebiotic fructo-oligosaccharide. The synbiotic powder was administrated after being dissolved in 1.5 mL of sterile water.

LPS, as a model bacterial endotoxin, was dissolved in 0.9% non-pyrogenic sterile NaCl (at a 10:1 ratio) and administered orally at a concentration of 250 μg/kg animal weight.

### Immunohistochemistry.

At the end of the experiment (on day 22), the mice were subjected to ether anaesthesia, after which the abdomen of Balb/c mice in both groups was opened. The ileum was cleaned and fixed in 10% formalin buffer solution, followed by dehydration, clearing and embedding. Tissue sections were probed with anti-mouse monoclonal antibodies against CD4 (C1805; Sigma-Aldrich) and CD8 (C7423; Sigma-Aldrich) to evaluate adaptive immune responses and an antibody against IgA (I6635; Sigma-Aldrich) to evaluate humoral immune responses. The samples were observed using a light microscope (CX21; Olympus, Tokyo, Japan) and photographed with an ILCE6000 camera (Sony, Tokyo, Japan). The number of immunopositive cells was determined by calculating the average number of cells in 20 random fields at magnification × 450 and expressed as the number of cells per field of vision.

### Statistical analysis.

Independent-samples *t*-test was used for normally distributed data and with Mann–Whitney U test for non-normally distributed data for differences between groups analysis. A value of p <0.05 was considered to be statistically significant.

## RESULTS

**Fig. 1:**
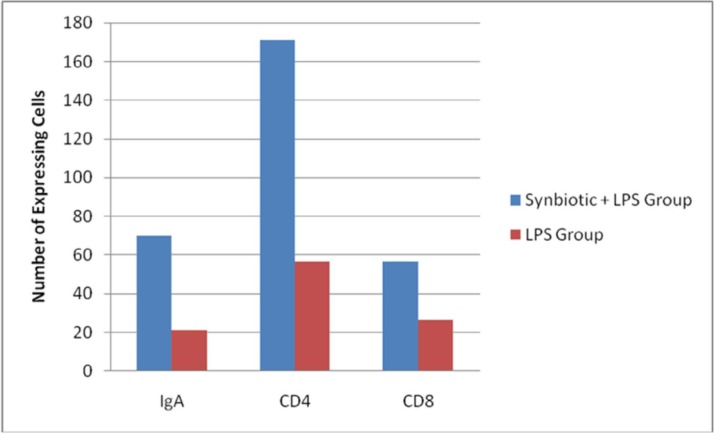
Mean numbers of IgA-, CD4- and CD8-expressing cells

The mean numbers of IgA-, CD4- and CD8-expressing cells in the K-I and K-II groups are presented in Fig. 1. Normality testing (Kolmogorov–Smirnov test) was first conducted as a prerequisite for analytic testing. The obtained data were then analysed using statistical tests (parametric). The results for IgA, CD4 and CD8 positivity were normally distributed in both groups, permitting parametric statistical tests to be performed.

Using an independent-samples *t*-test, the results revealed significant differences in the numbers of IgA- and CD4-expressing cells between the K-I and K-II groups (p = 0.027 and p = 0.009, respectively, Table 1). However, using the Mann–Whitney U test, the number of CD8-expressing cells did not differ between the groups (p = 0.199).

**Table 1.** Comparison of the numbers of IgA-, CD4- and CD8-expressing cells between the two groups

**Variables**	**Synbiotic + LPS Group**		**LPS Group**		**p value**
	
	**n**	**Mean (SD)**	**n**	**Mean (SD)**	
IgA	10	69.90 (57.93)	10	21.10 (12.31)	0.027
CD4	10	171.10 (107.14)	10	56.70 (47.37)	0.009
CD8	10	56.80 (56.03)	10	26.60 (9.25)	0.199

LPS, lipopolysaccharide

## DISCUSSION

A number of previous studies mentioned that synbiotics have the capacity to modulate innate and adaptive immune (both humoral and cellular) responses. The mechanism by which synbiotics modulate humoral immune responses is reflected by the production of immunoglobulin-producing cells (16). Immune responses induced by synbiotics in the intestinal flora mainly increase the numbers of IgA-producing cells without inducing systemic immune responses (16). In this study, the number of IgA-expressing, as a representation of humoral responses in the intestinal mucosa, was examined. Among all immunoglobulins, IgA-producing cells are predominant in number because they play a major role in limiting the penetration of antigens through the mucosal epithelium (17, 18).

LPS is an endoxin from Gram-negative bacteria that induces an inflammatory reaction; hence, the immunological resistance response will differentiate to Th1 and reduce the number of IgA-producing cells. Our results support this supposition. The observed high number of IgA-producing cells in K-I group is attributable to the activation of lymphocytes, which induce IgM to IgA switching (16). This result is also supported by a previous finding that probiotics could increase the numbers of both local and systemic IgA-producing cells (19). Oral LPS administration can increase the inflammatory response, stimulating the formation of pro-inflammatory cytokines and the balance towards the Th1 cell response (11, 12). LPS has been widely used for immunological experiments to illustrate the process of infection (13).

The protective effect of synbiotics against pathogens was also observed in comparisons of CD4+ and CD8+ cell counts. Our finding of higher numbers of CD4- and CD8-expressing cells in synbiotic-treated mice was similar to that of a previous study in which mucosal inflammation was decreased and the numbers of CD4+ and CD8+ cells in the lamina propria in mice were increased after the administration of *L. plantarum* (20). In addition, our findings with synbiotics are supported by another study involving 477 healthy human subjects who received probiotic supplements, which reported CD4+ and CD8+ cell counts compared with those in the placebo group (21).

This increase in the number of CD4-expressing cells in the K-I group was caused by the priming of the innate immune system in the intestinal mucosa by the synbiotic (22, 23). A previous study identified an increase in the number of CD4+ cells in the mesenteric lymph nodes in conventional mice administered LPS compared with the findings in LPS-treated germ-free mice. This finding illustrated that LPS and the gastrointestinal microbiota increase immune system function. As mentioned previously, oral probiotics stimulate mucosa immune cells to release pro-inflammation cytokines (24). This increase is caused by immune cell activation in the intestinal mucosa, particularly macrophages and dendritic cells involved in innate immunity (25).

LPS and probiotics are both components of extracellular bacteria, which could explain why combined administration of a synbiotic and LPS did not increase the number of CD8-expressing cells in this study. The same result was also obtained in another study in which germ-free and conventional mice were treated with different concentrations of LPS (26).

## CONCLUSION

In conclusion, the utilised multispecies synbiotic had immunoregulatory effects on IgA secretion and CD4+ cell counts, but not CD8+ cell counts, in LPS-exposed mice.
